# Regulated Inflammation and Lipid Metabolism in Colon mRNA Expressions of Obese Germfree Mice Responding to *Enterobacter cloacae* B29 Combined with the High Fat Diet

**DOI:** 10.3389/fmicb.2016.01786

**Published:** 2016-11-08

**Authors:** Huiying Yan, Na Fei, Guojun Wu, Chenhong Zhang, Liping Zhao, Menghui Zhang

**Affiliations:** State Key Laboratory of Microbial Metabolism, Joint International Research Laboratory of Metabolic and Developmental Sciences, School of Life Sciences and Biotechnology, Shanghai Jiao Tong UniversityShanghai, China

**Keywords:** mRNA, high-fat diet, B29, inflammation, lipid metabolism, obesity

## Abstract

Increased evidences have demonstrated that gut microbiota targeted diet intervention can alleviate obesity and related metabolic disorders. The underlying mechanism of interactions among diet, microbiota, and host still remains unclear. *Enterobacter cloacae* B29, an endotoxin-producing strain dominated in the gut of a morbidly obese volunteer (weight 174.8 kg, BMI 58.8 kg m^-2^) was isolated and transplanted to germfree mice (inoculated 10^10^ cells of B29 per day for 1 week). Using deep mRNA sequencing technology, we compared different gene expression profiles in the colon samples of the germfree mice treated with/without B29 and/or high fat diet (HFD) for 16 weeks and identified 279 differential expressed genes in total, including up-regulated genes *Apoa4* (fold change, 2.77), *Ido1* (2.66), *Cyp4a10* (7.01), and down-regulated genes *Cyp2e1* (0.11), *Cyp26b1* (0.34), *Akr1b7* (0.42), *Adipoq* (0.36), *Cyp1a1* (0.11), *Apoa1* (0.44), *Npc1l1* (0.37), *Tff2* (0.13), *Apoc1* (0.30*), Ctla2a* (0.34), *Mttp* (0.49), *Lpl* (0.48). Fifty-nine GO biological processes and five KEGG pathways, particularly the peroxisome proliferator-activated receptors signaling pathway, were significantly enriched in response to HFD+B29, which were mainly relevant to inflammation and the metabolism of lipid, lipoprotein, and sterols. These functional changes were consistent with the developed obesity, insulin-resistance, and aggravated inflammatory conditions of the HFD+B29 mice. This work provides insight into the gene expression changes in response to HFD+B29, helping to understand the mechanism of the interactions among HFD, B29 and the germfree mice.

## Introduction

The public health burden of obesity and metabolic disorders increases rapidly worldwide. The prevalence of these diseases is considered mainly due to the change of environmental factors, such as diet and lifestyles. Growing evidences have indicated that the gut microbiota is an important contributor to the development of these diseases (Turnbaugh et al., 2006; Backhed et al., 2007; Cani et al., 2008; Shen et al., 2013). Clinical trials and animal models have demonstrated that gut microbiota targeted diet intervention can alleviate obesity and related metabolic disorders.

In our previous clinical intervention trial, we found that a diet consisting of whole grains, traditional Chinese medicinal foods and prebiotics significantly reduced body weights and improved clinical parameters of volunteers with obesity (Fei and Zhao, 2013). Particularly, a volunteer (weight 174.8 kg, body mass index 58.8 kg m^-2^) had lost 51.4 kg and recovered from hyperglycemia and hypertension at the end of 23-week trial. Meanwhile, *Enterobacter*, a genus of opportunistic pathogens, decreased from 35% relative abundance of gut bacteria to non-detectable level. *Enterobacter cloacae* B29, an *Enterobacter* strain from this volunteer, was then isolated and transplanted to germfree mice. It was found that B29 combined with high fat diet (HFD) resulted in obesity and insulin resistance in germfree mice whereas B29 or HFD alone did not, confirming the causative role of B29 in metabolic deteriorations of the germfree mice (Fei and Zhao, 2013). As a LPS (lipopolysaccharide)-producing bacterium, B29 provided endotoxin load to the germfree mice that the B29+HFD group displayed the highest increase in systemic inflammation with the greatest increase in the serum amyloid A protein levels and the greatest decrease in adiponectin secretion. However, analysis with qRT-PCR (Reverse transcription-quantitative polymerase chain reaction) and microarray techniques found that the liver and the epididymal fat pad had significantly increased genes involved in inflammation while the ileum had not, suggesting the inflammation was local. The previous study did not investigate colon, which is an important immune organ, the main site of microbial fermentation with the highest bacterial concentrations and an active site of metabolism (Aura, 2008; Laparra and Sanz, 2010).

Many studies have tried to understand the underlying mechanism of how diet affects gut microbiota and the interactions between microbiota and the host through 16S rRNA based platform or metagenomic technology. The former is usually used to trace the change of the microbiota structure and the latter is to explore the function of the microbiome. In addition, some studies also have used microarray or/and qRT-PCR to investigate the gene expression of the host. However, both methods were restricted with the number and the sequencing depth of the genes detectable. A high throughput cDNA sequencing technology, mRNA-Seq, which is more powerful in characterizing transcript sequences and gene expressions, hence becomes an alternative candidate to provide more comprehensive information.

In this study, to comprehensively understand the mechanisms of the influences of B29 and HFD on the host, we collected the colon samples of these mice and measured the gene expressions using deep mRNA-Seq technology. We first compared the colon mRNA expression profiling of the four groups [Normal Chow Diet (NCD)+Luria-Bertani (LB), NCD+B29, HFD+LB, HFD+B29] and identified 279 differential expressed genes in total. The GO and KEGG enrichment analysis were then performed based on these differential expressed genes (DEGs) and regulations in inflammation and the metabolism of lipid, lipoprotein and sterols were observed. Finally, we picked the key DEGs involved in lipid and lipoprotein metabolism, inflammation and sterol metabolism under the stress of HFD+B29 and described how they respond to the provided stimuli.

## Materials and Methods

### Animal Experiment and Ethical Issues

Male C57BL/6J germfree mice purchased from Shanghai SLAC laboratory Animal Co., Ltd. (Shanghai, China) were raised in gnotobiotic isolators with a regular 12-h light cycle and supplied with sterile food and water. Before the age of 6–10 weeks, all mice were fed with a NCD and water *ad libitum*. Then they were randomly divided into four groups, each received one of the following treatments for 16 weeks: (1) NCD+LB group: inoculated 0.1 ml sterile LB medium per day for 1 week and fed with NCD; (2) NCD+B29 group: inoculated 10^10^ cells of B29 per mouse in 0.1 ml sterile LB medium per day for 1 week and fed with NCD; (3) HFD+LB group: inoculated 0.1 ml sterile LB medium per day for 1 week and fed with HFD; (4) HFD+B29 group: inoculated 10^10^ cells of B29 per mouse in 0.1 ml sterile LB medium per day for 1 week and fed with HFD (Fei and Zhao, 2013). At the end of week 16, all the 24 mice (*n* = 6/group) were killed by decollation. The colon tissues were collected for the mRNA sequencing, and the mass of epididymal fat pad, retroperitoneal fat pad, subcutaneous inguinal fat pad, and mesenteric adipose tissue were measured.

The experiment and relevant details were approved by the Institutional Animal Care and Use Committee (IACUC) of Shanghai Jiao Tong University, and were performed in accordance with relevant guidelines and regulations approved by the IACUC of Shanghai SLAC laboratory Animal Co., Ltd. All the potential biologically hazardous materials in this study were properly handled according to the Chinese biosafety laws and regulations.

### Total RNA Extraction and Purification

The total RNA from the colon tissues was extracted using TRIzol reagent (Invitrogen Life Technologies, Gaithersburg, MD, USA) and purified with RNeasy mini kit (QIAGEN, GmBH, Germany) and RNase-Free DNase Set (QIAGEN, GmBH, Germany), and the experiment was performed according to the manufacturer’s protocol. The integrity and quality of the total RNA were checked by a NanoDrop 1000 spectrophotometer (Thermo Scientific, Wilmington, DE, USA) and Agilent Bioanalyzer 2100 (Agilent technologies, Santa Clara, CA, USA), respectively. The ODA260/A280 of the RNA sample was between 1.9 and 2.1.

### Library Preparation and Sequencing

The RNA-seq library preparation was performed following the protocol recommended by the manufacturer (Illumina, San Diego, CA, USA). In brief, after purification, the mRNA was fragmented into small pieces, and the first-strand cDNA was prepared using random hexamers. Then the second-strand cDNA synthesis, end repair, addition of a single A base, and ligation of the adapters were performed. The products were purified and enriched by PCR to create the final cDNA library. The mRNA sequencing was done on the Illumina HiSeq 2500 with paired end 2 × 100 nt multiplex. The generated original sequence data can be downloaded from the NCBI GEO database (accession number: GSE87027).

### Bioinformatics and Statistics

The raw Illumina RNA-Seq reads were firstly processed to trim the adapter using Flexbar_v2.4_linux64 (Dodt et al., 2012), and to remove the reads shorter than 30 bp or with ‘*N*’ base using Prinseq-lite-0.20.3 (Schmieder and Edwards, 2011). The quality filtered reads were then mapped to the reference genome, the Mus_musculus genome ^1^ (Mus_musculus_Ensembl_NCBIM37.tar) by Tophat (version 2.0.10) (Trapnell et al., 2009) with default parameters, which allowed up to two mismatches.

The mapped sequences of each sample were assembled by Cuﬄinks (Trapnell et al., 2012b). The transcript abundance was estimated through incorporation of two normalization steps to ensure that the expression levels for different genes and the transcripts can be compared across runs, and was expressed as the unit of measurement fragments per kilobase of transcript per million mapped fragments (FPKM).

The obtained assembles were then merged by Cuffmerge (Trapnell et al., 2012b) with the reference annotation^1^ (Mus_musculus_Ensembl_NCBIM37.tar).

The differential expression analysis of the merged assembles was then performed with Cuffdiff (Trapnell et al., 2012b). Genes and transcripts whose |log2 (FoldChange)|≥ 1 and adjusted *p*-value < 0.05 (FDR adjusted with Benjamini–Hochberg) were picked as significant ones.

The enrichment analysis was performed with FIDEA^2^ (D’Andrea et al., 2013), and both up-regulated and down-regulated genes were considered. A threshold of 0.05 for *q*-value (FDR adjusted with Benjamini–Hochberg) was used to select significant enriched GO and pathway terms.

Gene counts of each sample were estimated by HTSeq-0.6.1p1 with the union mode assigning counts to the gene_id attribute^3^ (Anders et al., 2015), and the relative proportions of genes in each sample were calculated.

Canonical analysis and multivariate analysis of variance (MANOVA) clustering were performed under MATLAB^®^ R2010b environment.

The Venn diagram was drawn by Venny 2.1^4^. The bioinformatic protocols please refer to **Supplementary Figure S1.**

The information of the phenotypes related to the DEG was obtained from the Mouse Genome Informatics (MGI)^5^.

## Results

### B29+HFD Significantly Induced Obesity and Modulated the Colon mRNA Expression Profiling in Germfree Mice

Using Illumina Hiseq 2500 platform with paired end 2 × 100 nt multiplex sequencing, 3262.58 million raw reads in total of the 24 colon samples from the germfree mice were obtained. On average, 98.86% of the raw reads passed the quality control filtration (longer than 30 bp and without ‘*N*’ base), with a median of 129.79 million high quality reads per sample, ranged from 107.81 million to 180.17 million (Supplementary Table S1). The averaged overall mapping rate to the reference genome (the Mus_musculus genome) for the high-quality reads was 95.11% and the multiple alignments rate was 9.99% (Supplementary Table S2).

**FIGURE 1:**
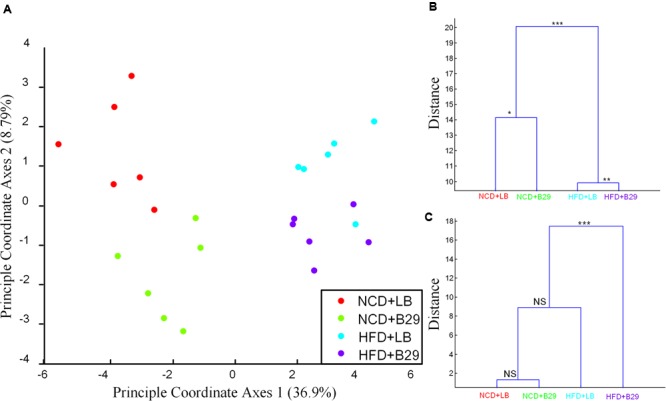
**The mRNA gene expression profiling and the physiological indexes of the four groups (NCD+LB, NCD+B29, HFD+LB, and HFD+B29) were affected by both high fat diet (HFD) and B29. (A)** Canonical analysis of the four groups based on the gene count matrix using eight PCs (accounting for 50.5% variation); **(B)** Multivariate analysis of variance (MANOVA) clustering based on the gene count matrix of the four groups using eight PCs (accounting for 50.5% variation); **(C)** MANOVA clustering based on the five physiological indexes (body weight, the mass of the fad pad of epididymis, retroperitoneum, subcutaneous inguinal, and mesenteric adipose tissue) of the four groups. ^∗∗∗^*p* < 0.001, ^∗∗^*p* < 0.01,^∗^*p* < 0.05.

Multivariate analysis of variance on the mRNA gene expression profiling in germfree mice showed that the four groups were significantly separated (**Figures 1A,B**). The HFD was the major factor contributing to the separation, and B29 was the secondary one. The gene expression compositions of the two groups fed with HFD (HFD+LB, HFD+B29) were significantly different from those of the other two groups with NCD (NCD+LB, NCD+B29; **Figure 1B**, MANOVA, *p* < 0.001). Furthermore, either within the HFD groups or the NCD groups, the group with B29 was significantly different from the group with LB, indicating that B29 also contributed to the change of the mRNAs (**Figure 1B**, MANOVA, HFD+LB vs. HFD+B29, *p* < 0.01; NCD+LB vs. NCD+B29, *p* < 0.05).

The differentiation among the four groups in phenotypes was somewhat different. According to Fei and Zhao (2013), at the end of the trial, the body weight, fat pad masses of epididymis, retroperitoneal, subcutaneous inguinal, and mesenteric adipose tissues of the mice were measured and we here used these five physiological indexes representing the targeted phenotype of the study. MANOVA showed that the phenotype of the NCD+B29 group was similar to that of the NCD+LB group, indicating the weak effect of B29 alone on phenotype under the NCD condition. Compared with the NCD+LB group (body weight 31.8 ± 1.2 g), the HFD+LB group had enhanced but not significantly changed body weight (34.4 ± 4.3 g, adjusted *p-*value > 0.05, Supplementary Table S3), which was consistent with the previous reports that germfree mice were resistant to HFD induced obesity (Turnbaugh et al., 2006; Backhed et al., 2007). Though these increased indexes caused the HFD+LB group being differentiated from the NCD+LB and the NCD+B29 group, no significant difference was observed among the three groups (**Figure 1C**). On the contrary, the HFD+B29 group had significantly higher body weight (45.4 ± 6.6 g, adjusted *p-*value < 0.01, Supplementary Table S3) and fat pad mass, making it be most differentiated and be significantly different from the rest three groups (**Figure 1C**, MANOVA, *p* < 0.001).

### Overall mRNA Gene Differential Expression Analysis

To facilitate the functional analysis of the mRNAs, the differential expressed mRNA genes were picked from the whole gene matrix with Cuffdiff (Trapnell et al., 2012a) according to the two standards: |log2 (FoldChange)|≥ 1 and adjusted *p*-value < 0.05. Compared with the NCD+LB control group, the NCD+B29, the HFD+LB, and HFD+B29 group had 135, 124, and 129 DEGs, respectively (**Table 1**, detailed DEGs were listed in Supplementary Table S4). Both the NCD+B29 and the HFD+LB group had much larger number of the up-regulated DEGs. While in the HFD+B29 group, the up- and down-regulated numbers were similar (65 vs. 64).

**Table 1:** The numbers of differential expressed genes (DEGs) from group comparisons.

Group Comparison	Number of DEG	Up-regulated genes	Down-regulated genes
A (NCD+LB) vs. B (NCD+B29)	135	117	18
A (NCD+LB) vs. C (HFD+LB)	124	96	28
A (NCD+LB) vs. D (HFD+B29)	129	65	64

**FIGURE 2:**
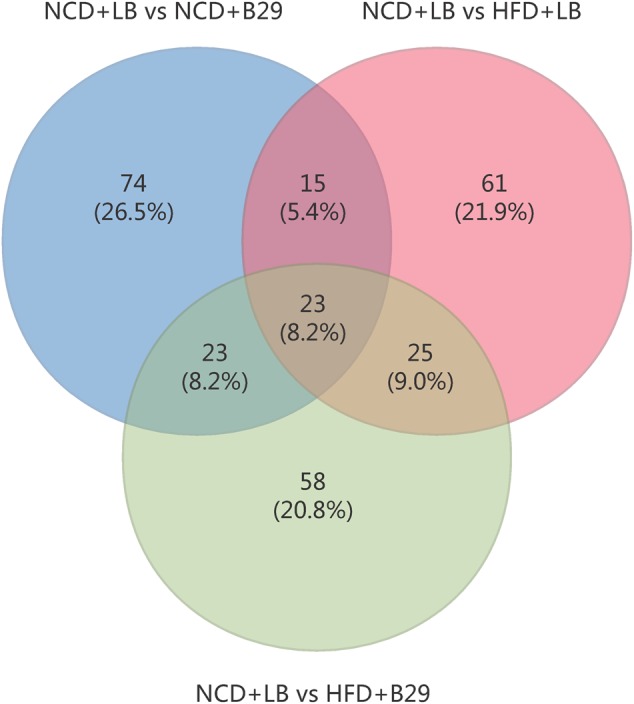
**Venn diagram of the differential expressed gene numbers from three comparisons (NCD+LB group vs. NCD+B29 group, NCD+LB group vs. HFD+LB group, NCD+LB group vs. HFD+B29 group, respectively)**.

Of the 279 non-redundant DEGs, only 23 were shared by the three groups (**Figure 2**). The group-specific ones occupied 69.2% of the total DEGs and almost distributed evenly among the three groups (the NCD+B29, HFD+LB, and HFD+B29 group had 74, 61, and 58 unique DEGs, respectively). This suggested that the HFD and B29 can affect the mRNA gene expression of the host colon either alone or together, but their influences might differ.

### GO and KEGG Pathway Enrichment Based on the Differential Expressed Genes

**FIGURE 3:**
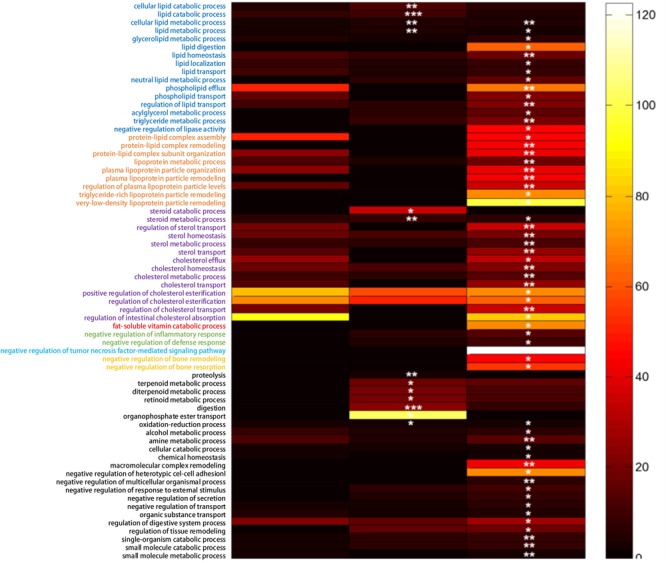
**The significant biological process GO terms enriched according to the obtained differential expressed genes (DEG) (FDR adjusted *q-*value < 0.05)**. The fold-change of each GO term enriched was represented by the color bar. ^∗∗∗^*p* < 0.001, ^∗∗^*p* < 0.01,^∗^*p* < 0.05.

According to the obtained 279 DEGs, the significantly enriched biological process GO terms were shown in **Figure 3**. Though there were 135 DEGs between the NCD+B29 group and the NCD+LB group, no biological process GO term was significantly enriched, suggesting that the 135 DEGs caused by B29 were relatively dispersive in biological process. Compared with the NCD+LB group, HFD+LB group had 13 enriched GO biological process terms, in particular four of which were related to lipid metabolism, and two to steroid metabolism. Significant changes were observed under the HFD+B29 condition that there were 59 enriched GO biological process terms, and particularly 55 of them appeared only when HFD and B29 co-existed. Within the 59 GO terms, 38 were of our interest since they were relevant to the phenotype of the germfree mice, of which 14 were related to lipid metabolism, 9 to lipoprotein metabolism, 13 to sterols metabolism and 2 to inflammatory response.

The GO enrichment analysis indicated that the effect of HFD or B29 alone to the host was relatively dispersive, whereas the joint effect of the two factors on the lipid, lipoprotein, sterols metabolism and inflammatory response of the host could be significantly enhanced. This was consistent with the phenotype changes of the mice during the animal trial that only the HFD+B29 group had significantly higher body weights, fat pad masses, triglyceride, and total cholesterol in serum than those of the other three groups (Fei, 2013).

The significant enriched KEGG pathways of the 279 DEGs were also obtained (**Table 2**). With 135 DEGs between the NCD+B29 group and the NCD+LB group, only two pathways were significantly enriched. One was the pathway of fat digestion and absorption, and the other was vitamin digestion and absorption. Unlike the result obtained by GO enrichment, the KEGG analysis indicated that B29 alone could increase fat digestion and absorption. The HFD+LB had five pathways enriched, including fat digestion and absorption, pancreatic secretion, protein digestion and absorption, retinol metabolism, and steroid hormone biosynthesis. This result was similar to that of the GO, suggesting that HFD alone could increase fat metabolism.

**Table 2:** The enriched KEGG pathway according to the obtained 279 differential expressed genes.

PATHWAY_ID	PATHWAY_NAME	A_B	A_C	A_D
mmu04975	Fat digestion and absorption	Enriched	Enriched	Enriched
mmu04977	Vitamin digestion and absorption	Enriched	NS	NS
mmu04972	Pancreatic secretion	NS	Enriched	NS
mmu04974	Protein digestion and absorption	NS	Enriched	NS
mmu00830	Retinol metabolism	NS	Enriched	Enriched
mmu00140	Steroid hormone biosynthesis	NS	Enriched	NS
mmu00980	Metabolism of xenobiotics by cytochrome P450	NS	NS	Enriched
mmu03320	PPAR signaling pathway	NS	NS	Enriched
mmu00380	Tryptophan metabolism	NS	NS	Enriched

*A: NCD+LB group; B: NCD+B29 group; C: HFD+LB group; D: HFD+B29 group.*

Using the DEGs between the HFD+B29 group and the NCD+LB group, five significantly enriched pathways were found, including fat digestion and absorption, retinol metabolism, metabolism of xenobiotics by cytochrome P450, peroxisome proliferator-activated receptor (PPAR) signaling pathway, tryptophan metabolism. The latter three pathways appeared only when HFD and B29 co-existed. PPARs are key nuclear receptor isoforms in the regulation of lipid and glucose metabolism, which play an important role in the regulation of obesity-related insulin sensitivity and inflammation. The significantly enriched PPAR signaling pathway only occurred to mice under the HFD+B29 condition, suggesting that the HFD and B29 should be combined together to cause the effect. Our previous qRT-PCR result obtained from the liver samples of the same mice also demonstrated that the PPAR_γ_ in HFD+B29 was markedly higher than those in the rest three groups (Fei, 2013). Hence, the enriched PPAR signaling pathway might be an important contributor to the obese, insulin-resistant, and inflammatory phenotypes of the corresponding HFD+B29 mice.

### Relevant Phenotypes and Functions of the Key DEGs under the Stress of HFD+B29

**FIGURE 4:**
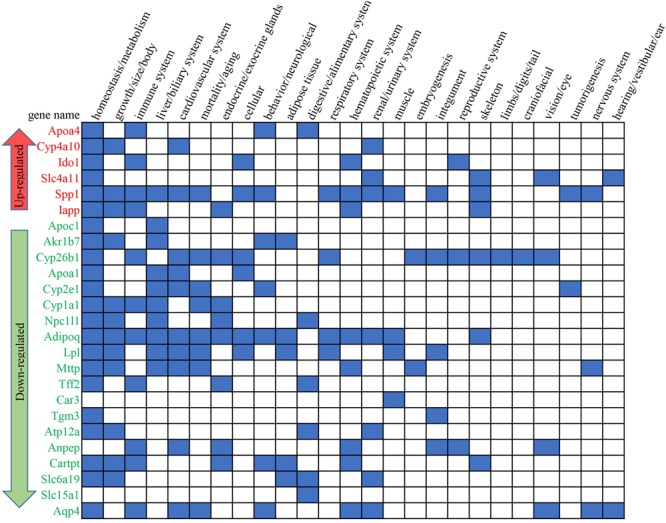
**The involved phenotypes of the key DEGs between the HFD+B29 group and the NCD+LB group.** The blue square means that relationship(s) existed according to the international database resource for the laboratory mouse mouse genome informatics (MGI).

From the 59 enriched GO terms under the stress of HFD+B29, 35 non-redundant DEGs of the HFD+B29 group were finally picked since many DEGs concurrently localized in different GO terms. These 35 DEGs were then put into the MGI (Eppig et al., 2015) the international database resource for the laboratory mouse, to search for their relevant phenotypes. As a result, 25 out of 35 DEGs were found to be related to known phenotypes and most of them had multiple relationships (**Figure 4**). These 25 key DEGs were annotated in **Table 3**. Of the 25 key DEGs, 6 were up-regulated and 19 were down-regulated. The obtained relevant phenotypes included homeostasis/metabolism (22 DEGs), growth/size/body (12 DEGs), immune system (11 DEGs), cardiovascular system (10 DEGs), liver/biliary system (10 DEGs), mortality/aging (8 DEGs), and endocrine/exocrine glands (8 DEGs). The phenotype searches also confirmed that the key DEGs were indeed relevant to the observed inflammation and obesity in HFD+B29 mice.

**Table 3:** The *q*-value, fold change and annotation of the key DEGs.

Gene Name	MGI_ID	Regulated	*q*-value	log2(fold change)	Annotations
Apoa4	88051	Up-regulated	0.014	1.47	Enzyme regulator, lipid binding, transporter, cell differentiation, cellular component organization, establishment of localization, homeostatic process, immune system process, lipid metabolic process, response to stimulus, system development
Cyp4a10	88611	Up-regulated	0.005	2.81	Oxidoreductase, lipid metabolic process
Ido1	96416	Up-regulated	0.005	1.41	Oxidoreductase, cell death, cell proliferation, immune system process, response to stimulus, system development
Slc4a11	2138987	Up-regulated	0.005	1.73	Transporter, establishment of localization, homeostatic process
Spp1	98389	Up-regulated	0.005	2.84	Receptor binding, cell death, cell differentiation, homeostatic process, immune system process, response to stimulus, system development
Iapp	96382	Up-regulated	0.010	1.76	Receptor binding, cell differentiation, homeostatic process
Apoc1	88053	Down-regulated	0.049	-1.72	Establishment of localization, lipid metabolic process
Akr1b7	101918	Down-regulated	0.005	-1.25	Oxidoreductase, lipid metabolic process
Cyp26b1	2176159	Down-regulated	0.005	-1.56	Lipid binding, oxidoreductase, cell death, cell differentiation, cellular component organization, homeostatic process, immune system process, lipid metabolic process, response to stimulus, signaling, system development
Apoa1	88049	Down-regulated	0.005	-1.17	Enzyme regulator, lipid binding, receptor binding, transporter, cell differentiation, cell proliferation, cellular component organization, establishment of localization, homeostatic process, immune system process, lipid metabolic process, protein metabolic process, response to stimulus, signaling, system development
Cyp2e1	88607	Down-regulated	0.005	-3.14	Oxidoreductase, lipid metabolic process, response to stimulus
Cyp1a1	88588	Down-regulated	0.005	-3.22	Oxidoreductase, lipid metabolic process, response to stimulus
Npc1l1	2685089	Down-regulated	0.005	-1.44	Cytoskeletal protein binding, transporter, establishment of localization, homeostatic process, lipid metabolic process
Adipoq	106675	Down-regulated	0.005	-1.48	Carbohydrate derivative binding, receptor binding, nucleic acid-templated transcription, cell deathcell differentiation, cell proliferation, cellular component organization, establishment of localization, homeostatic process, immune system process, lipid metabolic process, protein metabolic process, response to stimulus, signaling, system development
Lpl	96820	Down-regulated	0.005	-1.06	Carbohydrate derivative binding, hydrolase, lipid binding, receptor binding, cell differentiation, cellular component organization, establishment of localization, homeostatic process, lipid metabolic process
Mttp	106926	Down-regulated	0.032	-1.04	Lipid binding, transporter, cellular component organization, establishment of localization, homeostatic process, lipid metabolic process, protein metabolic process
Tff2	1306805	Down-regulated	0.005	-2.93	Receptor binding, cell proliferation, establishment of localization, immune system process, protein metabolic process, response to stimulus, signaling
Car3	88270	Down-regulated	0.005	-2.02	Hydrolase, response to stimulus
Tgm3	98732	Down-regulated	0.005	-3.21	Transferase, cell differentiation, cellular component organization, protein metabolic process, system development
Atp12a	1926943	Down-regulated	0.005	-1.16	Carbohydrate derivative binding, hydrolase, transporter, establishment of localization, homeostatic process, response to stimulus
Anpep	5000466	Down-regulated	0.005	-2.14	Hydrolase, cell differentiation, establishment of localization, homeostatic process, protein metabolic process, system development
Cartpt	1351330	Down-regulated	0.032	-1.21	Receptor binding, cell differentiation, establishment of localization, homeostatic process, immune system process, protein metabolic process, response to stimulus, signaling, system development
Slc6a19	1921588	Down-regulated	0.010	-1.05	Transporter, establishment of localization
Slc15a1	1861376	Down-regulated	0.005	-1.06	Transporter, establishment of localization
Aqp4	107387	Down-regulated	0.005	-1.14	Transporter, cellular component organization, establishment of localization, homeostatic process, immune system process, response to stimulus

**FIGURE 5:**
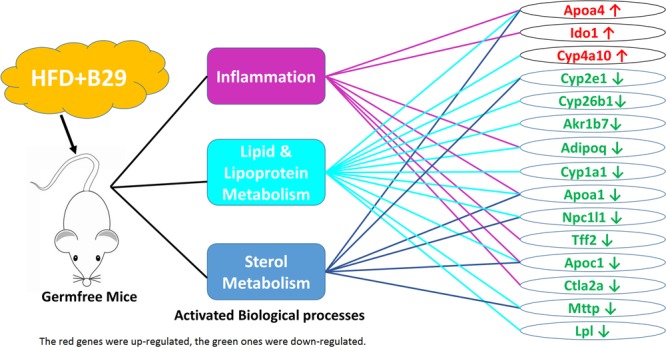
**The activated biological processes and their related DEGs under the stress of HFD+B29**.

Fifteen non-redundant DEGs of the HFD+B29 group were further picked out from the 38 GO terms involved in three biological process categories: inflammation, metabolism of lipid and lipoprotein, and metabolism of sterols (**Figure 5**). Three of them, *Apoa4, Ido1, and Cyp4a10* were up-regulated and 12 DEGs were down-regulated, including *Cyp2e1, Cyp26b1, Akr1b7, Adipoq, Cyp1a1, Apoa1, Npc1l1, Tff2, Apoc1, Ctla2a, Mttp, and Lpl*.

Among them, *Apoa4, Apoa1*, and *Apoc1* were related to the three biological processes simultaneously and *Cyp2e1, Adipoq, Npc1l1*, and *Mttp* were two function-related. *Ido1, Tff2*, and *Ctla2a* were only enriched in the inflammation related GO terms and *Cyp4a10, Cyp26b1, Akr1b7, Cyp1a1*, and *Lpl* were only enriched in the lipid and lipoprotein metabolism related GO terms. Genes involved in sterol metabolism related GO terms were all multiple function genes.

## Discussion

In this study, we used deep mRNA sequencing technology to investigate the different gene expression profiles under the stress with B29 and/or HFD. We found 279 DEGs in total were significantly regulated. Many DEGs had diverse functions, but mainly were related to inflammation, metabolism of lipid, lipoprotein and sterols. Particularly, three genes, *Apoa4, Apoa1*, and *Apoc1* were related to the three biological processes simultaneously. *Apoa4* had been reported to function in integrating feeding behavior, intestinal lipid absorption, and energy storage, and could improve glucose homeostasis by enhancing insulin secretion (Wang et al., 2012). Its production ApoA-4-containing lipoprotein was secreted by the small intestine in response to fat absorption, and was considered as an endogenous anti-inflammatory protein that could inhibit DSS-induced inflammation and colitis (Vowinkel et al., 2004), and could promote cholesterol eﬄux from cultured fibroblasts and adipose cells *in vitro* (Ordovas and Schaefer, 2000). The up-regulation of *Apoa4* in the HFD+B29 might be an irritable response in order to increase insulin secretion and decrease cholesterol load and inflammation level. While both *Apoa1* and *Apoc1* were down-regulated. *Apoa1* encoded apolipoprotein A1 (apoA1), the major protein component of high density lipoprotein (HDL), which played an important role in LPS neutralization and clearance (Guo et al., 2013), the reverse cholesterol transport process (Ordovas and Schaefer, 2000), and was reversely associated with the plasma level of HDL-Cholesterol (Gordon et al., 1977; Norum et al., 1982; Badimon et al., 1992; Srinivasan and Berenson, 1995). The absence of *Apoa1* could result in at least a 70% reduction in plasma HDL levels (Plump et al., 1997), which was a risk factor of atherogenic dyslipidemia and obesity. The down expression of the *Apoa1* in HFD+B29 might result in less LPS clearance and thus promote the level of endotoxin load and inflammation level and might be responsible for the increase of the plasma high-density lipoprotein cholesterol in the HFD+B29 mice (Fei, 2013). Another gene, *Apoc1* had been reported to increase the LPS-induced inflammatory state (Westerterp et al., 2007). The overexpression of human apolipoprotein C1 (Apoc1) in mice could protect mice from obesity and insulin resistance (Jong et al., 2001) whereas *Apoc1* deficient mice usually had decreased HDL-cholesterol levels (de Haan et al., 2008). The down-regulation of *Apoc1* in the HFD+B29 mice might be responsible for the occurred obesity, insulin resistance and higher plasma cholesterol level.

Four genes, *Cyp2e1, Adipoq, Npc1l1*, and *Mttp* were two biological processes-related genes, and all of them were down-regulated under the stress of HFD+B29. *Cyp2e1* was one of the major cytochrome P450 forms whose expression was strongly inhibited by inflammatory cytokines in humans and rodents (Hakkola et al., 2003), and was critically important in non-alcoholic steatohepatitis development by promoting oxidative/nitrosative stress, protein modifications, inflammation, and insulin resistance (Abdelmegeed et al., 2012). Alcohol-mediated up-regulation of *Cyp2e1* may initiate lipid peroxidation by the production of reactive oxygen species (Ekström and Ingelman-Sundberg, 1989; Ioannides, 1996; Lieber, 1997; Leclercq et al., 2000). The down-regulation of *Cyp2e1* in the HFD+B29 mice might be a result of the host high level of inflammation and be responsible for the observed fat accumulation. *Adipoq* encoded an adipose-derived hormone adiponectin (Scherer et al., 1995), which was suggested to have anti-inflammatory functions by stimulating a ceramidase activity, and was also a key modulator of lipid and glucose metabolism. The decreased adiponectin levels were correlated with chronic inflammation associated with obesity, type 2 diabetes, insulin resistance, and atherosclerosis (Berg et al., 2002; Hotamisligil, 2006; Hotamisligil and Erbay, 2008; Holland et al., 2011; Ouchi et al., 2011). The decreased serum adiponectin/body weight of the HFD+B29 in our previous animal experiment (Fei, 2013) might be related to the down-regulation of the *Adipoq*. *Npc1l1* was considered as a critical mediator of cholesterol absorption and functioned as a key modulator of whole-body cholesterol homeostasis (Davis et al. 2004, 2005, Jia, 2010). *Mttp* was critical for lipid and intestinal cholesterol absorption that its reduction might cause lower plasma lipid concentrations and intestinal deletions of *Mttp* could decrease plasma cholesterol concentrations by 45% (Iqbal et al., 2013). The decrease of *Npc1l1* and *Mttp* might be a host response to the enhanced levels of the plasma lipid induced by HFD+B29, and also might be responsible for the decrease of plasma cholesterol concentrations, which might be a response to the higher cholesterol level in the HFD+B29.

Three genes, *Ido1, Tff2*, and *Ctla2a* were only related to inflammation. As an important immune-regulator in the colon, *Ido1* was widely distributed in many tissues such as the gut (distal ileum and colon), lymph nodes, spleen, thymus, and lungs. Over-expression of *Ido1* had been reported under the exacerbation of colonic inflammation and could alter the production of inflammatory cytokine including *IFN-g, TNF-a, IL-10*, and *TGF-b* in colons (Takamatsu et al., 2013). The up-regulated *Ido1* in the colon under the stress of HFD+B29 might be a host response to the corresponding systemic inflammation. *Tff2* was a member of the trefoil factor family (TFF), playing a role in intestinal mucosal defense and repair, and in tumorigenesis (Leung et al., 2002). Human TFF2 could enhance the rate of colonic epithelial repair, and reduce local inflammation in a rat colitis model (Tran et al., 1999). The decreased expression of this gene in the HFD+B29 group might contribute to the enhanced level of the host inflammation. *Ctla2a* was an immunosuppressive factor and a cathepsin L (CathL) inhibitor that acted via retinal pigment epithelium to induce Treg cells (Sugita et al., 2009), which was reported to be up-regulated in the high-fat diet treated mice (Chen et al., 2005). However, it was down-regulated under the condition of HFD+B29.

Five genes, *Cyp4a10, Cyp26b1, Akr1b7, Cyp1a1*, and *Lpl* were only related to lipid and lipoprotein metabolism. *Cyp4a10* was a target gene of PPAR, which may play a significant role in fatty acid oxidation, and was usually increased expressed in obese mice (Enriquez et al., 1999). In wild-type mice, HFD was reported to be associated with a significant increase in hepatic *PPARα* mRNA and plasma free fatty acids, leading to a PPARα-dependent increase in expression of PPARα marker genes *Cyp4a10* (Kersten et al., 1999). The enhanced *Cyp4a10* level in this study, was consistent with the obese phenotype of the HFD+B29 mice. *Cyp26b1* was down-regulated in B29+HFD mice, and it was also reported to be down-regulated transcripts in obese-susceptible compared to lean-resistant subjects (Marrades et al., 2011). *Akr1b7* was a major regulator of white adipose tissue development through the inhibition of adipogenesis and lipogenesis, and lack of *Akr1b7* could increase basal adiposity and predispose to diet-induced obesity (Tirard et al., 2007; Volat et al., 2012). The down-regulation of *Akr1b7* might contribute to the obese phenotype of the HFD+B29 mice. The down-regulated *Cyp1a1* in the HFD+B29 mice was consistent with previous study that observed decreased expression of *Cyp1a1* in livers of morbidly obese women compared with massive weight loss women (Elam et al., 2009). *Lpl* was related to the hydrolysis of triglycerides in plasma lipoproteins and generation of fatty acids (Olivecrona and Olivecrona, 2009; Wang and Eckel, 2009). Suppressed expression of *Lpl* in pancreatic beta cells was observed in obese mice (Nyrén et al., 2012). According to these reports, the down-regulation of these four genes was consistent with the occurrence of the obese phenotype of the host.

The PPAR signaling pathway was enriched based on the DEGs only when HFD and B29 co-existed. A recent study reported that high-fat diet modified the PPAR_γ_ pathway leading to disruption of microbial and physiological ecosystem in murine small intestine (Tomas et al., 2016). Our previous qRT-PCR result obtained from the liver samples of the same mice also demonstrated that the PPAR_γ_ in HFD+B29 was markedly higher than those in the rest three groups (Fei, 2013). These results suggested that the expression of the PPAR signaling pathway in different tissues could be simultaneously regulated by high-fat diet. In addition, the PPAR pathway could not be regulated when germfree mice were only treated with HFD in our study, indicating that B29 was important and essential for HFD to regulate the PPAR pathway.

Our previous results had reported that the expression of the inflammation related genes *TNFα, IL-1β, IL-6, IKK-𝜀, TLR4* increased significantly in the liver and epididymal fat pad but not in the ileum of the HFD+B29 gnotobiotic mice, Thus, the previous study supposed that there were local inflammation induced in the former two tissues but not in the gut (Fei, 2013). However, in this study, the significantly increased expression of inflammation-involved genes in the colon gave opposite evidences. The changed functions indicated by the mRNAs in the colon cell were consistent with the enhanced serum LBP, total cholesterol, triglycerides, and the decreased serum adiponectin/body weight of the HFD+B29 mice, which suggested that the inflammation indeed occurred in the gut and that the influence of HFD and B29 on the inflammation and lipid metabolic were systemic. The previous study could not observed inflammation in the ileum might due to two reasons, one is that different parts of the gut (such as ileum and colon) might have different immune responses to the stress HFD+B29, and the other might be due to the limited information obtained with qRT-PCR, which only have several genes of the ileum be checked. The lack of detection depth in qRT-PCR used in the ileum samples further implied the importance and power of mRNA technology.

Both the HFD and B29 could independently and largely change the mRNA gene expressions as many DEGs were identified. However, these DEGs were relatively dispersive that few relevant enriched GO terms or KEGG pathways were observed under HFD or B29 alone. This may explain why HFD or B29 alone could not significantly cause the change in body weight and fat pad masses in the animal trial. The inconsistence between results from the mRNA profiles and the phenotype in mice implied that the difference in gene composition may not enough to reflect the corresponding difference in function. Investigation of the mRNAs at functional level is a better way to associate the change of the mRNAs with that in phenotypes.

The significantly enhanced inflammatory response, the metabolism of lipid, lipoprotein, and sterols under the HFD+B29 condition suggested the synergistic effect of the HFD and B29, though it was hard to deduce whether B29 could facilitate HFD to cause effect or vice versa. However, the information obtained from the mRNA profiles helped to understand why only the HFD+B29 mice developed obesity, got insulin-resistant phenotype, and aggravated inflammatory conditions.

This comparative study provided insight into the gene expression changes corresponding to the stimuli of HFD+B29, helping to understand the interactions among the HFD, B29 and the germfree mice, and the phenotype consequences caused by HFD+B29.

## Author Contributions

NF and LZ designed research; NF and CZ performed experimental procedure; HY, MZ, and GW performed data analysis and annotations; HY, LZ, and MZ wrote the paper.

## Conflict of Interest Statement

The authors declare that the research was conducted in the absence of any commercial or financial relationships that could be construed as a potential conflict of interest.

## Footnotes

1 http://support.illumina.com/sequencing/sequencing_software/igenome.html

2 http://www.biocomputing.it/fidea

3 http://www-huber.embl.de/users/anders/HTSeq/

4 http://bioinfogp.cnb.csic.es/tools/venny/index.html

5 http://www.informatics.jax.org/

## Supplementary Material

FIGURE S1 **The bioinformatic protocols**.
