# Supplementary figures and images for: Regulated Inflammation and Lipid Metabolism in Colon mRNA Expressions of Obese Germfree Mice Responding to Enterobacter cloacae B29 Combined with the High Fat Diet
Source: Front Microbiol. 2016 Nov 8;7:1786. doi: 10.3389/fmicb.2016.01786 (PMC5099522; doi:10.3389/fmicb.2016.01786)

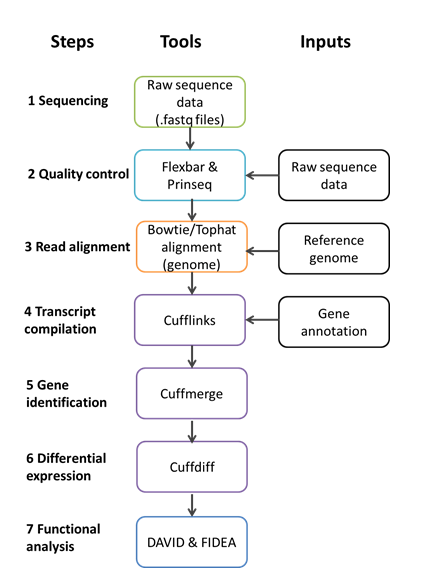

Supplement: FIGURE S1 — The bioinformatic protocols. [file Image_1.TIF]
