# Supplementary material for: Regulated Inflammation and Lipid Metabolism in Colon mRNA Expressions of Obese Germfree Mice Responding to Enterobacter cloacae B29 Combined with the High Fat Diet
Source: Front Microbiol. 2016 Nov 8;7:1786. doi: 10.3389/fmicb.2016.01786 (PMC5099522; doi:10.3389/fmicb.2016.01786)
Supplement: Supplementary file 2 [file Table_1.DOCX]

***Supplementary Material***

**Regulated inflammation and lipid metabolism in colon mRNA expressions of obese germfree mice responding to *Enterobacter cloacae* B29 combined with the high fat diet**

**Huiying Yan, Na Fei, Guojun Wu, Chenhong Zhang, Liping Zhao, Menghui Zhang ***

State Key Laboratory of Microbial Metabolism, Joint International Research Laboratory of Metabolic & Developmental Sciences, and School of Life Sciences and Biotechnology, Shanghai Jiao Tong University, Shanghai, 200240, P.R.China

*** Correspondence:**Corresponding Author:Menghui Zhang
[mhzhang@sjtu.edu.cn](mailto:mhzhang@sjtu.edu.cn)

**Supplementary Tables**

**Supplementary Table 1.** Sequencing information of the 24 colon samples.

| Sample ID | Raw reads (10^6) | QC reads (10^6) | QC ratio | Q20 Value | Q30 Value |
| --- | --- | --- | --- | --- | --- |
| A001 | 138.24 | 135.95 | 98.35% | 96.73% | 90.27% |
| A002 | 122.92 | 121.06 | 98.48% | 96.84% | 90.45% |
| A003 | 155.66 | 151.73 | 97.48% | 95.67% | 87.23% |
| A004 | 141.44 | 140.29 | 99.19% | 97.12% | 90.68% |
| A005 | 121.43 | 120.40 | 99.16% | 97.05% | 90.58% |
| A006 | 116.91 | 115.80 | 99.05% | 96.96% | 90.38% |
| B001 | 170.98 | 166.50 | 97.38% | 95.56% | 86.99% |
| B002 | 160.29 | 157.95 | 98.54% | 97.10% | 91.25% |
| B003 | 136.95 | 135.78 | 99.15% | 97.19% | 91.07% |
| B004 | 126.92 | 125.79 | 99.11% | 96.99% | 90.45% |
| B005 | 118.95 | 117.85 | 99.07% | 96.93% | 90.29% |
| B006 | 116.28 | 115.24 | 99.10% | 96.93% | 90.29% |
| C001 | 145.96 | 144.03 | 98.68% | 96.36% | 88.86% |
| C002 | 151.12 | 149.73 | 99.08% | 96.91% | 90.22% |
| C003 | 129.94 | 128.75 | 99.09% | 96.89% | 90.23% |
| C004 | 116.19 | 115.20 | 99.15% | 96.93% | 90.17% |
| C005 | 121.24 | 120.15 | 99.11% | 96.93% | 90.23% |
| C006 | 120.77 | 119.72 | 99.14% | 97.03% | 90.50% |
| D001 | 162.14 | 160.85 | 99.20% | 97.13% | 90.73% |
| D002 | 111.86 | 110.89 | 99.13% | 97.02% | 90.53% |
| D003 | 109.07 | 107.81 | 98.84% | 96.84% | 90.22% |
| D004 | 153.37 | 152.00 | 99.11% | 97.07% | 90.65% |
| D005 | 181.98 | 180.17 | 99.01% | 96.96% | 90.50% |
| D006 | 132.00 | 130.82 | 99.11% | 97.07% | 90.71% |

A: NCD+LB group; B: NCD+B29 group; C: HFD+LB group; D: HFD+B29 group.
