# Supplementary material for: Regulated Inflammation and Lipid Metabolism in Colon mRNA Expressions of Obese Germfree Mice Responding to Enterobacter cloacae B29 Combined with the High Fat Diet
Source: Front Microbiol. 2016 Nov 8;7:1786. doi: 10.3389/fmicb.2016.01786 (PMC5099522; doi:10.3389/fmicb.2016.01786)
Supplement: Supplementary file 3 [file Table_2.DOCX]

***Supplementary Material***

**Regulated inflammation and lipid metabolism in colon mRNA expressions of obese germfree mice responding to *Enterobacter cloacae* B29 combined with the high fat diet**

**Huiying Yan, Na Fei, Guojun Wu, Chenhong Zhang, Liping Zhao, Menghui Zhang ***

State Key Laboratory of Microbial Metabolism, Joint International Research Laboratory of Metabolic & Developmental Sciences, and School of Life Sciences and Biotechnology, Shanghai Jiao Tong University, Shanghai, 200240, P.R.China

*** Correspondence:**Corresponding Author:Menghui Zhang
[mhzhang@sjtu.edu.cn](mailto:mhzhang@sjtu.edu.cn)

**Supplementary Tables**

**Supplementary Table 2**. The number of reads sequenced and mapped with Tophat

| Sample ID | Total reads | Overall Mapping Rate | multiple alignments | discordant alignments |
| --- | --- | --- | --- | --- |
| A001 | 135949048 | 92.60% | 3939834 (6.4%) | 988392 (1.6%) |
| A002 | 121056970 | 94.60% | 5295393 (9.4%) | 777961 (1.4%) |
| A003 | 151733762 | 93.90% | 6175657 (8.9%) | 872658 (1.3%) |
| A004 | 140291786 | 96.40% | 7401640 (11.1%) | 763860 (1.1%) |
| A005 | 120402844 | 96.20% | 5595659 (9.8%) | 785000 (1.4%) |
| A006 | 115795244 | 96.50% | 5497878 (10.0%) | 786172 (1.4%) |
| B001 | 166504476 | 95.10% | 7286471 (9.4%) | 1128717 (1.5%) |
| B002 | 157951004 | 95.80% | 5886124 (7.9%) | 1154984 (1.6%) |
| B003 | 135784116 | 90.60% | 6521485 (10.8%) | 1187954 (2.0%) |
| B004 | 125786392 | 96.10% | 5795146 (9.8%) | 738881 (1.2%) |
| B005 | 117847726 | 94.80% | 5363418 (9.8%) | 703310 (1.3%) |
| B006 | 115239968 | 94.60% | 6201178 (11.6%) | 738090 (1.4%) |
| C001 | 144033678 | 91.90% | 15115318 (23.4%) | 956026 (1.5%) |
| C002 | 149726206 | 96.20% | 7715279 (10.9%) | 963461 (1.4%) |
| C003 | 128752678 | 95.90% | 5721931 (9.4%) | 861749 (1.4%) |
| C004 | 115198506 | 96.40% | 4614976 (8.5%) | 748700 (1.4%) |
| C005 | 120153380 | 95.70% | 4957056 (8.8%) | 736967 (1.3%) |
| C006 | 119721816 | 95.00% | 5410868 (9.7%) | 745706 (1.3%) |
| D001 | 160848200 | 95.90% | 6576199 (8.7%) | 836126 (1.1%) |
| D002 | 110885576 | 95.80% | 4834896 (9.3%) | 608081 (1.2%) |
| D003 | 107806010 | 93.70% | 4700370 (9.5%) | 647224 (1.3%) |
| D004 | 151997892 | 96.00% | 6998513 (9.8%) | 890075 (1.2%) |
| D005 | 180173674 | 96.40% | 7960546 (9.3%) | 1197944 (1.4%) |
| D006 | 130818022 | 96.60% | 4674142 (7.5%) | 814290 (1.3%) |

A: NCD+LB group; B: NCD+B29 group; C: HFD+LB group; D: HFD+B29 group.
