# Supplementary material for: Regulated Inflammation and Lipid Metabolism in Colon mRNA Expressions of Obese Germfree Mice Responding to Enterobacter cloacae B29 Combined with the High Fat Diet
Source: Front Microbiol. 2016 Nov 8;7:1786. doi: 10.3389/fmicb.2016.01786 (PMC5099522; doi:10.3389/fmicb.2016.01786)
Supplement: Supplementary file 4 [file Table_3.DOCX]

***Supplementary Material***

**Regulated inflammation and lipid metabolism in colon mRNA expressions of obese germfree mice responding to *Enterobacter cloacae* B29 combined with the high fat diet**

**Huiying Yan, Na Fei, Guojun Wu, Chenhong Zhang, Liping Zhao, Menghui Zhang ***

State Key Laboratory of Microbial Metabolism, Joint International Research Laboratory of Metabolic & Developmental Sciences, and School of Life Sciences and Biotechnology, Shanghai Jiao Tong University, Shanghai, 200240, P.R.China

*** Correspondence:**Corresponding Author:Menghui Zhang
[mhzhang@sjtu.edu.cn](mailto:mhzhang@sjtu.edu.cn)

**Supplementary Table 3**. Physiological indexes of the germfree mice.

| Physiological indexes | NCD+LB | NCD+B29 | HFD+LB | HFD+B29 |
| --- | --- | --- | --- | --- |
| Body weight(g) | 31.8±1.2 | 30.4±1.6 | 34.4±4.3 | 45.4±6.6(**)(△△△)(##) |
| Epididymal fat pad(g) | 0.311±0.035 | 0.346±0.041 | 1.141±0.333(***) | 2.343±0.495(***)(△△) (###) |
| Retroperitoneal fat pad(g) | 0.064±0.021 | 0.074±0.030 | 0.399±0.121 | 1.180±0.429(***)(△△) (##) |
| Subcutaneous inguinal fat pad(g) | 0.167±0.048 | 0.208±0.045 | 0.638±0.166 | 1.193±0.831(***)(△△) (##) |
| Mesenteric adipose tissue(g) | 0.115±0.012 | 0.199±0.081 | 0.382±0.073 | 1.125±0.516(***)(△△) (#) |

* represented the adjusted P value compared with the control group NCD+LB, ***p<0.001, **p<0.01,*p<0.05

△ represented the adjusted P value of group HFD+B29 compared with group NCD+B29, △△△p<0.001, △△p<0.01, △p<0.05

# represented the adjusted P value of group HFD+B29 compared with group HFD+LB, ###p<0.001, ##p<0.01, #p<0.05

Data were provided by Na Fei.
