# Supplementary material for: Regulated Inflammation and Lipid Metabolism in Colon mRNA Expressions of Obese Germfree Mice Responding to Enterobacter cloacae B29 Combined with the High Fat Diet
Source: Front Microbiol. 2016 Nov 8;7:1786. doi: 10.3389/fmicb.2016.01786 (PMC5099522; doi:10.3389/fmicb.2016.01786)
Supplement: Supplementary file 5 [file Table_4.DOCX]

***Supplementary Material***

**Regulated inflammation and lipid metabolism in colon mRNA expressions of obese germfree mice responding to *Enterobacter cloacae* B29 combined with the high fat diet**

**Huiying Yan, Na Fei, Guojun Wu, Chenhong Zhang, Liping Zhao, Menghui Zhang ***

State Key Laboratory of Microbial Metabolism, Joint International Research Laboratory of Metabolic & Developmental Sciences, and School of Life Sciences and Biotechnology, Shanghai Jiao Tong University, Shanghai, 200240, P.R.China

*** Correspondence:**Corresponding Author:Menghui Zhang
[mhzhang@sjtu.edu.cn](mailto:mhzhang@sjtu.edu.cn)

**Supplementary Tables**

**Supplementary Table 4**. The list of differential expressed genes.

| A(NCD+LB) vs B(NCD+B29) | A(NCD+LB) vs C(HFD+LB) | A(NCD+LB) vs D(HFD+B29) |
| --- | --- | --- |
| **Up-regulated** | **Up-regulated** | **Up-regulated** |
| Cd28 | Aqp12 | Rgs13 |
| Ctse | 1810030J14Rik | Creb3l3 |
| Cacna1s | U6 | SNORA33 |
| Rgs16 | Prdx6 | SNORA17 |
| 1810030J14Rik | Gm8540 | AC120540.1 |
| Gpr55 | Creb3l3 | Serpina3k,Serpina3m,Serpina3n |
| Prdx6 | Upp1 | Ighv3-6 |
| Fcrla | Ccl8 | Ighv6-3 |
| Gm8540 | G6pc | Ighv1-18 |
| Upp1 | 9130204K15Rik | Ighv1-39 |
| Tnfrsf13b | E130012A19Rik | Ighv1-53 |
| SNORA33 | Krt17 | Ighv1-64 |
| Slc46a1 | Ppy | Ighv8-12 |
| Hoxb13 | Pyy | Ighv1-72 |
| 9130204K15Rik | Slc26a3 | Ighv1-76 |
| Ighg1 | Ighv3-5 | Ighv1-80 |
| Ighv9-1,Ighv9-3 | Ighv1-19 | Ighv1-85 |
| Ighv3-6 | Ighv1-22 | Hist1h2bq |
| Ighv3-8 | Ighv1-26 | Slc17a4 |
| Ighv6-3 | Ighv8-8 | SNORA17 |
| Ighv6-6 | Ighv1-64 | Pcdh17 |
| Ighv10-1 | Ighv1-80 | Ang4 |
| Ighv1-4,Ighv1-7 | Ighv1-85 | Pcdh17 |
| Ighv1-12,Ighv1-9 | Akr1c18 | AC154683.1 |
| Ighv1-15 | Hist1h2bq | SNORA70 |
| Ighv1-18 | Hist1h2ap | Gsdmc |
| Ighv1-19 | Atp12a | Gsdmc2 |
| Ighv1-22 | SNORA40 | Gsdmc3 |
| Ighv1-26 | Pcdh17 | Gsdmc4 |
| Ighv1-34 | Rnase1 | Ly6a,Ly6c1 |
| Ighv1-39 | Ang4 | 9030619P08Rik |
| Ighv1-52 | Gsdmc | Mir3470b |
| Ighv1-53 | Gsdmc2 | Iglc2 |
| Ighv1-55 | Gsdmc3 | Cst6 |
| Ighv1-64 | Gsdmc4 | Hck |
| Ighv1-66 | 9030619P08Rik | Slc4a11 |
| Ighv8-12 | Cela1 | U6 |
| Ighv1-72 | Tfrc | Reg4 |
| Ighv1-76 | Retnlb | Sprr1a |
| Ighv1-80 | Iglc3,Iglv1 | Cyp4a10 |
| Ighv1-81 | Iglc2 | Gm12574 |
| Ighv1-85 | SNORA17 | Gnat3 |
| Hist1h2bq | Clps | Spp1 |
| Thbs4 | Tff2 | Cyp3a44 |
| Gzma | Crisp1,Crisp3 | n-R5s160 |
| Atp12a | Srd5a2 | Igkv1-110,Igkv1-117 |
| Gzmb | Cyp2c66 | Igkv16-104 |
| Slc15a1 | Pnliprp1 | Igkv15-103 |
| BC025446,Ly6g | Pnliprp2 | Igkv3-2 |
| Higd1c.1,Mettl7a2,RP24-73K9.3.1 | 2010003K11Rik | Iapp |
| Gsdmc | Cst6 | Igkv10-94,Igkv10-96 |
| Gsdmc2 | Ankrd1 | Igkv4-59 |
| Gsdmc3 | Cyp2c69 | Igkv4-55 |
| Gsdmc4 | Scd1 | Siglec5 |
| Ly6d | Slc27a2 | Psg28 |
| 9030619P08Rik | Rbpjl | 9830147E19Rik |
| Iglc3,Iglv1 | Cel | n-R5s97 |
| Iglc2 | Gm10800 | Ces2a |
| Iglv2 | Reg4 | Ido1 |
| Csta | Pitx2 | Apoa4 |
| Psmb8 | Cpb1 | Gm16869 |
| Capn13 | Sprr1a | Gm10680 |
| Srd5a2 | Spink4 | **Down-regulated** |
| 2010001M09Rik | Orm1 | Gsta3 |
| Spink3 | Cyp4a10 | U6 |
| Cyp2c67,Cyp2c68 | AL805897.1 | H60c |
| SNORA44 | Gm12574 | L3mbtl3 |
| Hoxd13 | Insl5 | Trpv2 |
| Slc20a1 | Cela2a,Ctrc | Hoxb13 |
| Tgm3 | Dmp1 | U8 |
| Pla2g4f | Spp1 | Npc1l1 |
| Rims4 | Oas3 | Slc36a2 |
| Reg4 | Cyp3a44 | A530016L24Rik |
| Adh1 | Cpa2 | Ighv2-6-8,Ighv2-9-1 |
| Fam46c | Prss2 | Ighv5-17 |
| Gm11830 | Sval1,Sval3 | Ighv1-47 |
| Ggh | Igkv14-100 | Gm1574 |
| Insl5 | Reg1 | Ctla2a |
| U6 | 2210010C04Rik | Slc6a19 |
| Pla2g5 | Erp27 | Cartpt |
| Areg | Sycn | Atp12a |
| Igj | 2410002F23Rik | U4 |
| Cyp3a44 | Aqp8 | Slc15a1 |
| Sval1,Sval3 | Gm15495 | Cyp2d12,Cyp2d9 |
| AI854703 | Gp2 | Adipoq |
| Tnip3 | Ctrl,Psmb10 | Retnla |
| Igkv2-137 | Tmed6 | Cbr3 |
| Igkv14-111,Igkv14-126,Igkv9-120,Igkv9-129 | Sf3b3 | Msln |
| Igkv1-110,Igkv1-117 | Ctrb1 | Tff2 |
| Igkv15-103 | Apoa4 | Aqp4 |
| Igkv14-100 | Rbp2 | snoU13 |
| Igkv3-10,Igkv3-3,Igkv3-4,Igkv3-5,Igkv3-7 | Gm16869 | Tgm3 |
| Igkv3-2 | Slc37a2 | Ankrd5,BC034902 |
| Igkc | Gm10680 | Mal |
| Reg3b | U5 | Car3 |
| Aldh1l1 | mt-Nd2 | Gbp1 |
| Clec4d | **Down-regulated** | Sis |
| Igkv10-94,Igkv10-96 | SNORA17 | Mttp |
| Igkv19-93 | H60c | SNORA2 |
| Igkv4-74 | U6 | Gm12913 |
| Igkv4-72 | Trpv2 | Insl5 |
| Igkv4-68 | U8 | Upk3b |
| Igkv4-59 | Ighv1-47 | Akr1b7 |
| Igkv4-57-1 | Ighv1-75 | Sval1,Sval3 |
| Igkv4-57 | Ctla2a | Igkv1-135 |
| Igkv4-55 | Cartpt | Reg3b |
| Cyp2f2 | Gzma | Reg3a |
| Scnn1g | U4 | Aldh1l1 |
| Aqp8 | Naaladl1 | Inmt |
| Gm15495 | Gbp1 | Igkv4-61 |
| Neu3 | Sis | Cyp26b1 |
| Gp2 | Mttp | 2610206C17Rik,AC099599.1 |
| Ces2a | Upk3b | SNORA17 |
| Ido1 | Igkv2-137 | Cyp2e1 |
| Best2 | Reg3b | Apoc1 |
| Pou2af1 | Cyp26b1 | Anpep |
| Gm17322 | SNORA17 | 3100003L05Rik |
| **Down-regulated** | Anpep | n-R5s158 |
| SNORA17 | n-R5s158 | Lpl |
| U6 | Bmper | Apoa1 |
| U8 | Cyp1a1 | Cyp1a1 |
| Ang4 | 2010106E10Rik | Lars2 |
| U4 |  | Glb1l2 |
| snoU13 |  | U5 |
| Mttp |  | 2010106E10Rik |
| SNORA2 |  |  |
| AI747448 |  |  |
| Igkv1-135 |  |  |
| 2610206C17Rik,AC099599.1 |  |  |
| SNORA17 |  |  |
| n-R5s158 |  |  |
| AC160635.1 |  |  |
| Apoa1 |  |  |
| Apoa4 |  |  |
